# Consortia of bioactives in supercritical carbon dioxide extracts of mustard and small cardamom seeds lower serum cholesterol levels in rats: new leads for hypocholesterolaemic supplements from spices

**DOI:** 10.1017/jns.2019.28

**Published:** 2019-09-24

**Authors:** Soumi Chakraborty, Kaninika Paul, Priyanka Mallick, Shrabani Pradhan, Koushik Das, Saikat Chakrabarti, Dilip Kumar Nandi, Paramita Bhattacharjee

**Affiliations:** 1 Department of Food Technology and Biochemical Engineering, Jadavpur University, Kolkata 700032, India; 2 Division of Structural Biology and Bioinformatics, CSIR-Indian Institute of Chemical Biology, 4, Raja S.C. Mullick Road, Kolkata 700032, India; 3 Research Unit developed by Department of Physiology, Nutrition and Microbiology, Raja N.L. Khan Women's College, Midnapore 721102, West Bengal, India

**Keywords:** Supercritical carbon dioxide extraction, Yellow mustard seeds, Small cardamom seeds, Hypocholesterolaemic activity, HMG-CoA reductase, AI, atherogenic index, BW, body weight, EDX, energy dispersive X-rays, EPR, electron paramagnetic resonance, FT-IR, Fourier transformed IR spectroscopy, GOLD, Genetic Optimization for Ligand Docking, HDL-C, HDL-cholesterol, HED, human equivalent dose, HMG-CoA, 3-hydroxy-3-methyl-glutaryl-CoA, LC-ESI-MS, liquid chromatography-electrospray ionisation MS, LDL-C, LDL-cholesterol, OECD, Organization of Economic Co-operation and Development, PDB, protein data bank, SC, small cardamom, SC_best_, small cardamom seed extract obtained at the optimum conditions of SC-CO_2_, SC-CO_2_, supercritical CO_2_, TC, total cholesterol, YM, yellow mustard, YM_best_, yellow mustard seed extract obtained at the optimum conditions of SC-CO_2_

## Abstract

Melatonin-rich and 1,8-cineole-rich extracts have been successfully obtained from yellow mustard (YM) and small cardamom (SC) seeds, respectively, employing green technology of supercritical CO_2_ (SC-CO_2_) extraction. Chemical profiling confirmed the presence of melatonin and 1,8-cineole and co-extractants in the respective extracts. Electron paramagnetic resonance spectroscopy attested strong antioxidant activities of the extracts foregoing pan-assay interference compounds involved in spectroscopic analysis. These extracts also exhibited synergistic efficacies greater than unity confirming antioxidant synergy among the co-extracted bioactives therein. To ascertain hypocholesterolaemic efficacies, these extracts were co-administered orally with Triton X (at the pre-optimised dose of 175 mg/kg body weight (BW)) to Wistar albino rats at doses of 550, 175 and 55 mg/kg BW. Serum total cholesterol levels in the rats were monitored on days 3, 7, 15 and 21. On day 21, total cholesterol level reduced appreciably by 49·44 % in rats treated with YM seed extract and by 48·95 % in rats treated with SC seed extract, comparable with atorvastatin-administered rats (51·09 %). Either extract demonstrated inhibitory effects on hepatic 3-hydroxy-3-methyl-glutaryl-CoA (HMG-CoA) reductase activity. A molecular docking exercise identified specific compounds in the extracts which possessed binding affinities comparable with therapeutically used HMG-CoA reductase inhibitors. *In silico* and *in vivo* studies concertedly concluded that the consortium of bioactive components in the extracts cannot be considered as invalid metabolic panaceas and therefore these ‘green’ extracts could be safely subjected to clinical studies as preventive biotherapeutics for hypercholesterolaemia. These extracts could be consumed *per se* as hypocholesterolaemic supplements or could be ingredients of new spice-based therapeutic foods.

The seeds of spice crops yellow mustard (*Brassica campestris*), commonly known as ‘field mustard’, and small cardamom (*Elettaria cardamomum*), the ‘Queen of spices’, are consumed as culinary condiments in India and also across the globe. These spices are reportedly known to possess strong antioxidant, anti-inflammatory and antimicrobial properties^(1,2)^. It has been demonstrated that consumption of 3 g whole cardamom per d has antihypertensive, anti-inflammatory and antioxidant activities in humans^(3,4)^. However, there is no documented evidence on similar therapeutic benefits of whole mustard seeds in humans.

Despite noteworthy benefits of the above seeds, consumption of the whole seeds *per se* and also as constituents (seasonings) of cooked foods for exploitation of the benefits of ‘food synergy’^(5)^ is limited owing to their appreciably high Scoville heat unit values and thermal degradation of their constituent heat-sensitive bioactives. To forego these limitations, we have adopted the ‘reductionism’ approach^(5)^ to obtain ‘bioactive-rich extracts’ from these spices by employing the green technology of supercritical CO_2_ (SC-CO_2_) extraction. The extraction parameters have been optimised (by statistical design of experiments and analysis) to yield extracts rich in their respective bioactive principles, namely melatonin-rich extract from yellow mustard (YM) seeds^(6)^ and 1,8-cineole-rich extract from small cardamom (SC) seeds^(7)^. These extracts have exhibited *in vitro* inhibition of cholesterol solubility in micellar solution (60·34–84·56 %)^(6,7)^, which indicates possible inhibition of intestinal cholesterol absorption^(8)^.

Bisson *et al*.^(9)^ have recommended that it is crucial to verify the bioactivity of compounds before designating them as ‘authentic natural leads’. Therefore, the above extracts have been subjected to rigorous *in vitro* chemical profiling and assessment of their antioxidative efficacies including synergism among the co-extracted antioxidants. All these *in vitro* findings prompted us to explore the efficacies of the aforesaid extracts in redressing *in vivo* hypercholesterolaemia in Wistar albino rats. Rat models have been selected owing to their physiological, anatomical and genetic similarities to human beings^(10)^. All *in vivo* studies were conducted in accordance with Organization of Economic Co-operation and Development (OECD) guidelines.

Triton X-100 is reportedly the classical cholesterol-inducing drug^(11,12)^. Although the use of Triton X-100 in different doses in inducing hypercholesterolaemia in rats has been documented by several authors^(12–15)^, the rationale behind the choice of the said doses is not reported, implying non-specific selection of doses by researchers. Moreover, it is not apparent from the literature whether these randomly selected doses could achieve a stable hypercholesterolaemic condition in the experimental rats. This necessitated thorough optimisation of the dose of Triton X-100 in the present study to first achieve a stable hypercholesterolaemic condition in the Wistar rats to enable unambiguous judgement of the hypocholesterolaemic efficacies of the spice extracts thereafter. The extracts were then co-administered with the optimised dose of Triton X-100 in rats to investigate their efficacies as preventive medicines for hypercholesterolaemia.

Therefore, the specific objectives of the present study are: (a) chemical profiling of the extracts by Fourier transformed IR spectroscopy (FT-IR), GC-MS and liquid chromatography-electrospray ionisation MS (LC-ESI-MS); (b) evaluation of *in vitro* antioxidative efficacies in terms of their radical-scavenging potencies and synergistic effects (SE) among the co-extracted antioxidants; (c) optimisation of dose and duration of Triton X-100 administration to induce a stable hypercholesterolaemic condition in Wistar rats with minimum toxicity effects on liver and kidney tissues; (d) to investigate the hypocholesterolaemic efficacies in terms of their lipid profiles and atheroprotective efficacies; and (e) investigation of the inhibitory effects of the extracts on 3-hydroxy-3-methyl-glutaryl-CoA (HMG-CoA) reductase (the key indicator of cholesterol biosynthesis pathway) activity.

It is envisaged that these spice extracts would primarily act as inhibitors of HMG-CoA reductase and thereby lower serum total cholesterol (TC) levels, and/or would exhibit a secondary effect by scavenging reactive oxygen species produced during hypercholesterolaemia and therefore mitigate *in vivo* lipid peroxidation in the animals. Although few *in vivo* studies exist on efficacies of mustard (*Brassica juncea*) and small cardamom (*E. cardamomum*) seeds in redressing hypercholesterolaemia in animal models^(16,17)^, only changes in lipid profiles of the animals have been reported.

The present study is the first systematic detailed study in accordance with OECD guidelines for the elucidation of hypocholesterolaemic efficacies of SC-CO_2_ extracts of SC and YM seeds. Additionally, molecular docking studies were performed by docking the major extract constituents with HMG-CoA reductase, followed by comparative evaluation of their binding potentials with a standard statin-based drug. This exercise allowed us to identify which chemical compounds among the major extracted constituents inhibited HMG-CoA reductase activity. The findings of *in silico* and *in vivo* studies were then corroborated to eliminate ambiguity in conferring these spice extracts as novel biotherapeutics. The outcome of the present study can safely be extrapolated to redress hypercholesterolaemia in humans. These extracts would serve as hypocholesterolaemic supplements or could be ingredients for new spice-based therapeutic foods or drugs.

## Materials and methods

### Materials

The authenticated B_9_ species of YM seeds (*B. campestris*) were provided by IRDM and authenticated Alleppey green cardamom (*E. cardamomum*) seeds (of export quality) were procured from Spices Board. Specialty chemicals such as melatonin (99 % pure), 1,8-cineole (99 % pure) and Triton X-100 (99 % pure) were procured from Sigma. The pure standards of α-terpineol, α-terpinyl acetate, linalool and limonene were gifted by M/s Keva Flavours Pvt Ltd (Mumbai, India). Food-grade CO_2_ was purchased from BOC India Ltd. An SPE-ED matrix for SFE vessel packing and an SPE-ED SPE cartridge were procured from Applied Separations. All the chemicals for biochemical tests were collected from Merck Specialities Pvt. Ltd and HiMedia Laboratories Pvt. Ltd.

### Supercritical carbon dioxide extraction of ayurceuticals: melatonin from yellow mustard and 1,8-cineole from small cardamom seeds

**Fig. 1.**
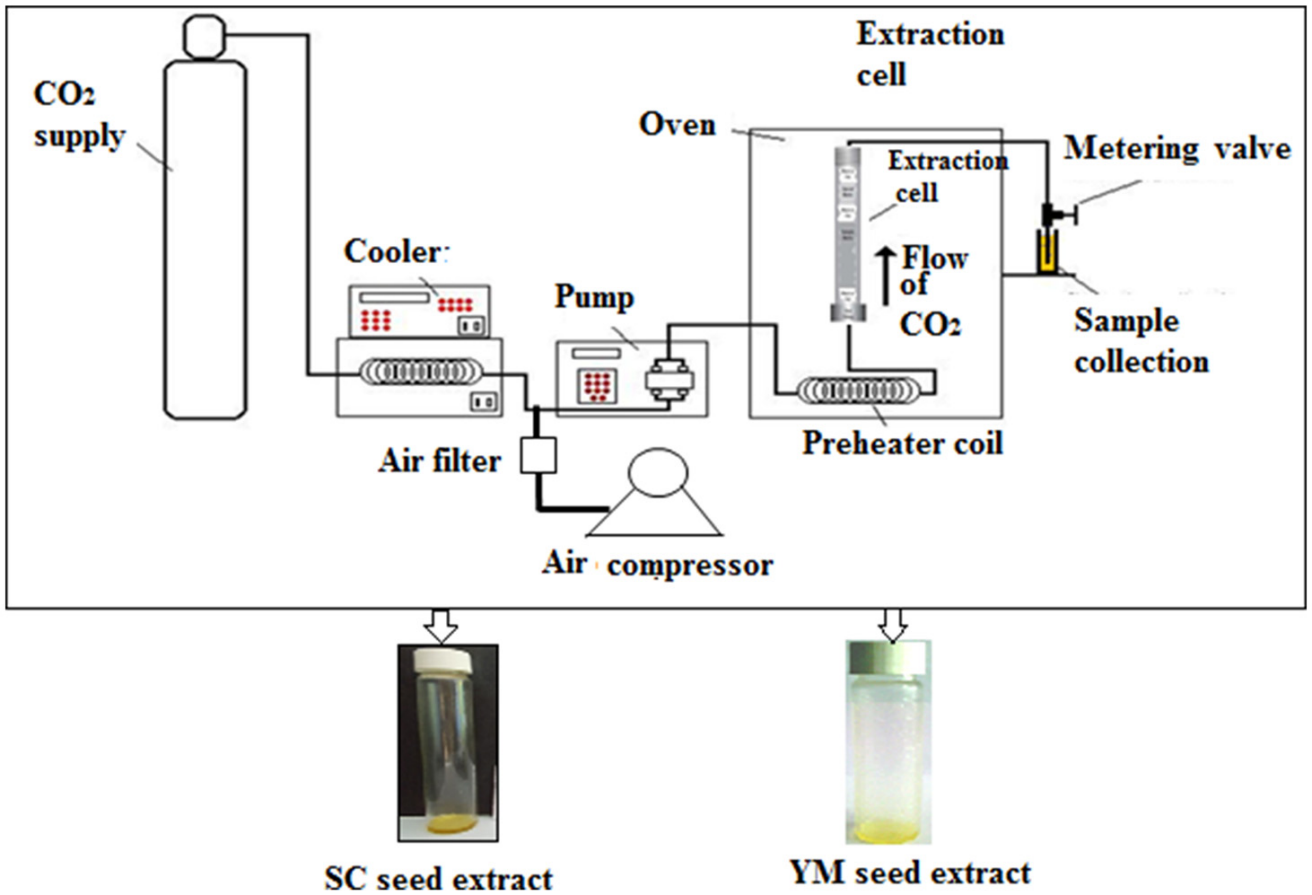
Schematic diagram of the SPE-ED SFE unit of Applied Separations, USA. SC, small cardamom; YM, yellow mustard.

A laboratory scale ‘SCF Green Technology SPE-ED SFE 2 model’ (Applied Separations) was used for SC-CO_2_ extractions of melatonin and 1,8-cineole from YM and SC seeds, respectively^(6,7)^ (Fig. 1). The optimised conditions that provided the maximum yield of melatonin were a sample size of 30 g (*d*_p_ = 0·5 ± 0·01 mm) of YM seeds at a pressure of 300 bar at 50°C and 120 min extraction time at a flow rate of 2 litres/min of gaseous CO_2_^(6)^. The optimised conditions that provided the maximum yield of 1,8-cineole were a sample size of 25 g (*d*_p_ = 0·5 ± 0·01 mm) of SC seeds at a pressure of 200 bar, 50°C, 90 min extraction time and a flow rate of CO_2_ = 2 litres/min^(7)^. The extracts of YM and SC seeds obtained at the optimum conditions of SC-CO_2_ were designated as SC_best_ and YM_best_, respectively.

### Chemical profiling of SC_best_ and YM_best_

#### Quantification of melatonin in YM_best_ and 1,8-cineole in SC_best_

Melatonin in YM_best_ was quantified by HPLC according to the method reported by Chakraborty & Bhattacharjee^(6)^ while quantification of 1,8-cineole in SC_best_ was conducted densitometrically using high-performance TLC (HPTLC) in accordance with the method described by Ghosh *et al*.^(1)^. Both HPLC and HPTLC chromatograms suggested the presence of other co-extractants in the extracts in addition to their active principles (discussed later).

#### Fourier transformed IR analyses of SC_best_ and YM_best_ extracts

FT-IR analyses of SC_best_ and YM_best_ were performed to preliminarily identify the co-extracted compounds present in the extracts. The extracts were placed on KBr pellets and subjected to a Spectrum 100 FT-IR spectrometer (PerkinElmer) with a light source of silicon carbide and frequency range of 1000–4000/cm. The peaks of the FT-IR spectra were matched with available FT-IR spectra (Spectrabase™ database) of the other compounds present in YM_best_ (namely tocopherol, ascorbic acid, limonene and linalool, oleic acid) and SC_best_ (namely α-terpinyl acetate, linalool, limonene and α-terpineol) extracts.

#### Identification of co-extractants in the YM_best_ and SC_best_

YM_best_ and SC_best_ were filtered using a 0·22 µm syringe filter and then subjected to LC-ESI-MS and GC-MS, respectively, for identification of the co-extracted antioxidants. YM_best_ was diluted with 10 % dimethyl sulfoxide (DMSO) and subjected to LC-ESI-MS analysis in accordance with the method reported by Halder *et al*.^(18)^, with a few modifications. The LC system (Shimadzu Corporation) equipped with a binary pump (LC-20AD), an autosampler (SIL-20A), an online solvent degasser (DGU-20A3) and column oven (CTO-10AS) were used in this study. An aliquot (10 µl) of diluted sample was injected into a C18 analytical column (Phenomenex Kinetex, 50 mm × 3 mm, internal diameter 5 µm). Mass spectrometric detection was performed in the multiple reactions monitoring mode.

SC_best_ was analysed using GC-MS to detect the presence of co-antioxidants therein in accordance with the method reported by Ghosh *et al*.^(1)^, with a few modifications. A Perkin Elmer GC Clarus 680 chromatograph (Perkin Elmer) equipped with a fused silica capillary column (30 × 0·25 mm internal diameter, 0·25 µm film thickness) coupled to a Perkin Elmer Clarus SQ 8T mass Detector (Perkin Elmer) was employed for the analyses. Identification of the components of the extracts was based on matching the mass spectra with those available in the NIST (2000) library of compounds and from literature reports of mass spectra by Adams and others^(19–21)^.

#### Quality assessment of YM_best_ and SC_best_ in terms of their antioxidant efficacies

*2,2-Diphenyl-1-picrylhydrazyl (DPPH) radical scavenging activity:* The antioxidant activities of YM_best_ and SC_best_ were primarily evaluated by DPPH radical scavenging activity employing the spectrophotometric method^(22)^ and expressed as IC_50_ (50 % radical scavenging activity of DPPH) values. However, there is possibility of obtaining false-positive absorbance^(9)^ in this spectrophotometric assay owing to pan-assay interference compounds (PAINS). Therefore, the above extracts were further subjected to electron paramagnetic resonance (EPR) spectroscopic analysis for validation of their radical-scavenging activities.

*Electron paramagnetic resonance analysis of extracts to eliminate the interference of ‘pan-assay interference compounds’ (PAINS):* To eliminate PAINS in the above assay, the extracts were subjected to EPR spectroscopic analysis in accordance with the method reported by Chakraborty & Bhattacharjee^(6)^.

*Assessment of antioxidant synergism in the extracts in terms of SE values:* The antioxidants (discussed later) present in YM_best_ and SC_best_ may impede the antioxidative function of either melatonin or 1,8-cineole, if they do not act in synergism. Hence, *in vitro* synergism among 1,8-cineole, α-terpinyl acetate, limonene, α-terpineol and linalool in SC_best_ and that among melatonin, tocopherol, ascorbic acid, limonene and linalool in YM_best_ were determined in terms of SE values in accordance with the method described by Chakraborty & Bhattacharjee^(23)^.

#### Safety assessment of YM_best_ and SC_best_ in terms of heavy metals and *in vivo* acute toxicity

Since solventless SC-CO_2_ extractions of YM_best_ and SC_best_ did not necessitate further downstream processing and purification, the extracts thus obtained were analysed for the presence of heavy metals, if any. Also, *in vivo* acute toxicities of these were determined in Wistar albino rats to ensure safe oral administration thereafter.

### Presence of heavy metal toxicants

Energy dispersive X-ray (EDX) analyses of YM_best_ and SC_best_ were performed in an EDX spectrometer (JSM-6700F; JEOL Ltd) to detect the presence of toxic metals such as Ti, Cu, Pb, Hg, As, Zn, Se, Ni, Mo and Si, if any. The analyses were carried out at 20 kV using the JEOL detector under high vacuum (25–27 mbar).

### *In vivo* acute toxicity study

As per OECD guidelines 423^(24)^, acute toxicities of SC-CO_2_ extracts (YM_best_ and SC_best_) were determined in terms of their LD_50_ values using the limit dose of 5000 mg of extract/kg body weight (BW). Wellness parameters of animals were observed and recorded systematically after 30 min, 1 h, 2 h, 3 h, 4 h, 24 h and once regularly up to day 14, post-administration of the said dose. The wellness parameters included changes in skin, fur, eyes and mucus membrane along with respiratory problems, changes in behavioural pattern, tremor, convulsions, salivations, diarrhoea, lethargy, sleep and mortality.

### Evaluation of *in vivo* hypocholesterolaemic activities of YM_best_ and SC_best_

**Fig. 2.**
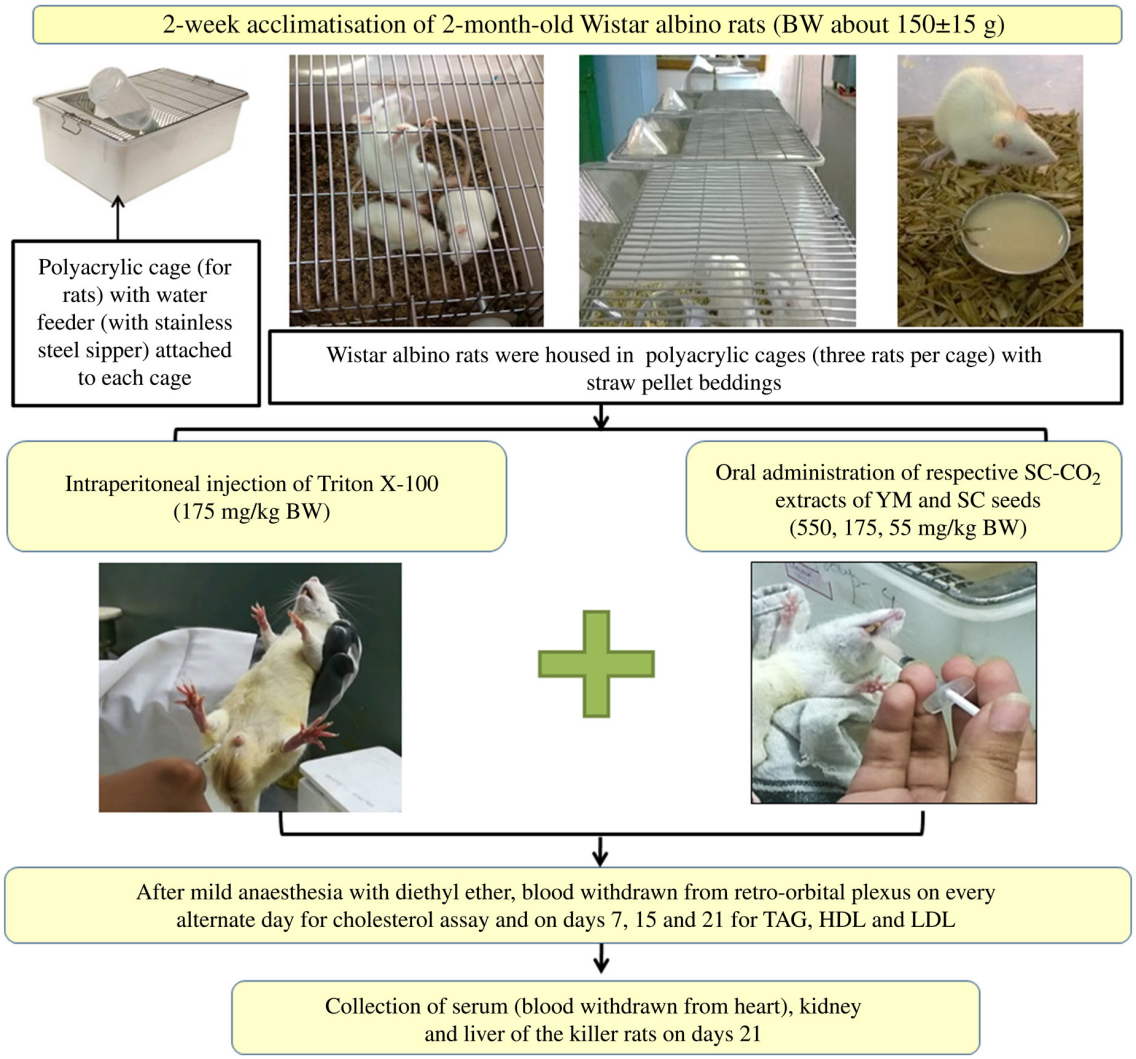
Schematic representation of the *in vivo* experimental study. BW, body weight; SC-CO_2_, supercritical carbon dioxide; YM, yellow mustard; SC, small cardamom.

**Fig. 3.**
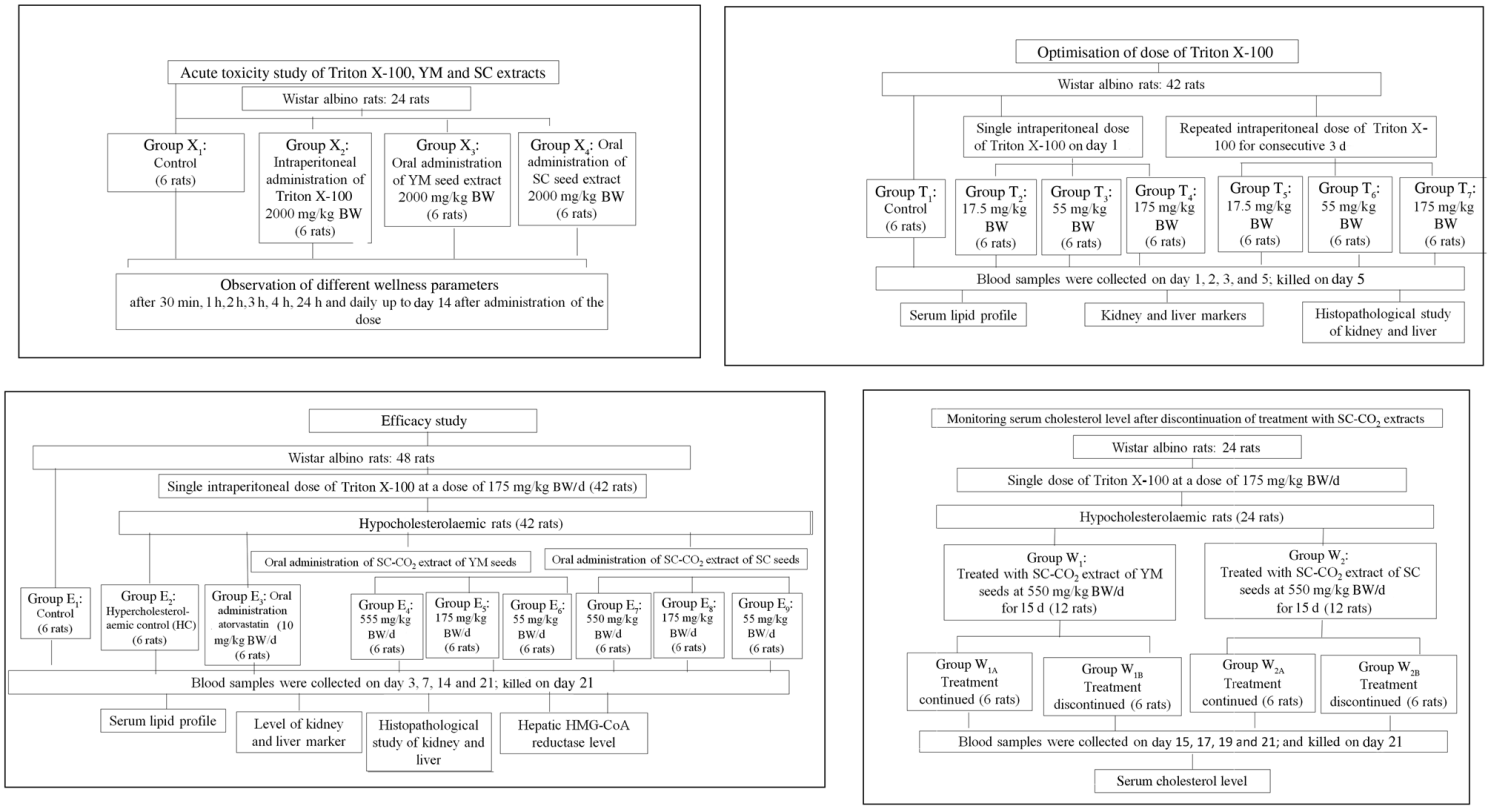
Experimental design of the *in vivo* hypocholesterolaemic study. YM, yellow mustard; SC, small cardamom; BW, body weight; SC-CO_2_, supercritical carbon dioxide; HC, healthy controls; HMG-CoA, 3-hydroxy-3-methyl-glutaryl-CoA.

Prior to co-administration of the extracts in Wister albino rats, the dose and duration of Triton X-100 administration were optimised to induce fast and stable hypercholesterolaemia in the animals. Groups of rats and details of *in vivo* experiments are schematically presented in Figs 2 and 3.

### Experimental animal acclimatisation and welfare-related assessments

Male Wister albino rats (130–150 g) aged 2 months were procured from Rita Ghosh Private Ltd. The study was conducted in the laboratory of the Department of Physiology, Nutrition and Microbiology, Raja N.L. Khan Women's College, Midnapore, West Bengal, India, in the months of January to March 2017. The experiments were conducted as per approved guidelines of Institutional Animal Ethical Committee (IAEC) guidelines and CPCSEA (Committee for the Purpose of Control and Supervision of Experiments on Animals; registration no. 190/PO/Re/S/2016/CPCSEA).

The animals were maintained and acclimatised under standard environmental conditions (17–23°C, 60 ± 5% relative humidity) for 3 weeks prior to the study. Acclimatised rats were selected randomly and three rats per polyacrylic cage (30 × 23 × 10 cm) were housed inside a temperature- and humidity-controlled room with 12 h dark–12 h light (180–200 lux at day time) cycles. They were provided with standard rat feed (a mixture of wheat flour, Bengal gram flour, milk powder, salt and distilled water, on fresh w/w basis) and distilled water *ad libitum* throughout the experimental period. Animal care and personal hygiene of the researchers were maintained according to the *Guide to the Care and Use of Experimental Animals*^(25)^. Food intake and body weight were measured regularly during the experimental period.

### Acute toxicity study of Triton X-100

Prior to standardisation of the dose of Triton X-100, an acute toxicity study of Triton X-100 was conducted in accordance with the method described for assessment of toxicity of the extracts.

### Standardisation of dose of Triton X-100 for inducing hypercholesterolaemia

After overnight fasting, rats were injected intraperitoneally (Fig. 2) with 17·5, 55 and 175 mg/kg BW doses of Triton X-100 prepared in 10 % dimethyl sulfoxide (DMSO) solution, in accordance with a dose progression factor of 3·2 times as per OECD guidelines 425^(26)^ (Fig. 3). Triton X-100 was administered in the morning at 09.00 hours. To induce fast and stable hypercholesterolaemia, the rats were injected intraperitoneally both in ‘single’ doses of Triton X-100 at 17·5, 55 and 175 mg/kg BW and in ‘multiple’ doses (i.e. each dose of 17·5, 55 and 175 mg/kg BW administered daily for 3 consecutive days).

Blood was withdrawn from the retro-orbital plexus of the rats on days 1, 2, 3 and 5 (same time on each day) after mild anaesthesia with diethyl ether-soaked cotton. Since there were insignificant changes in TC levels of the rats on days 3 and 5, post-injection of a single dose of Triton X-100 on day 1 (discussed later), the rats were killed on day 5. Heart blood collected from killed rats was centrifuged at 2000 ***g*** for 10 min, the serum was collected and analysed for TC^(27)^, TAG^(28)^, HDL-cholesterol (HDL-C)^(29)^ and LDL-cholesterol (LDL-C)^(30)^ levels using their respective standard kits. Hypercholesterolaemia in the rats was confirmed when serum cholesterol level remained stable at 200–210 mg/dl (5·18–5·44 mmol/l)^(31)^.

### Co-administration of YM_best_ and SC_best_ with Triton X-100

Hypercholesterolaemia was induced in the rats by injecting an optimised dose (discussed in the Results and Discussion sections) of Triton X-100 (175 mg/kg BW), intraperitoneally. Extracts were co-administered to the rats orally (a reliable method for administering substances into the gastrointestinal tract) at concentrations of 550, 175 and 55 mg/kg BW, selected using the dose progression factor 3·2^(26)^, after 1 h of single-dose Triton injection on day 1 and at 10.00 hours from day 2 onwards (Fig. 3). Blood was collected from the retro-orbital plexus of rats on days 3, 7, 15 and 21 (after mild anaesthesia with diethyl ether). It was observed that there were insignificant changes in the TC levels of the rats on days 15 and 21 (discussed later); the rats were therefore killed on day 21.

### Monitoring serum total cholesterol level after discontinuation of oral administration of YM_best_ and SC_best_

The optimised dose and duration of administration of YM_best_ and SC_best_ (obtained from above experiment) were found to be 550 mg/kg BW and 15 d, respectively (details of optimisation have been described later). Since there were negligible changes in serum TC levels of the extract-administered rats on days 15 and 21, the serum cholesterol levels of rats were monitored on days 15, 17, 19 and 21 (by withdrawing blood from the retro-orbital plexus of the rats) after discontinuation of extract administration on day 15 to ascertain whether the same remained unaltered up to day 21. Concomitantly, the wellness (physical and/or psychological) parameters of the rats were also monitored.

### Withdrawal of blood samples and collection of organs for biochemical and histological assays

On day 21, the rats were kept in a fasting condition overnight and killed by cervical dissipation on the following day. Blood was withdrawn from their aortas by cardiac puncture. The kidneys and livers of the animals were also recovered implementing standard dissection procedures^(32)^ of histological analyses.

### Biochemical estimation of plasma lipid profile and atherogenic indices

The above collected serum samples (post-scarification) were subjected to TAG, HDL-C and LDL-C analyses on day 21 according to the above-mentioned standard methods. Serum VLDL-cholesterol level and atherogenic index (AI) were calculated according to Mallick *et al*.^(33)^ and Namani *et al*.^(34)^, respectively.

### Spectrophotometric determination of hepatic 3-hydroxy-3-methyl-glutaryl-CoA reductase activity

Liver samples were prepared in accordance with a reported method^(35)^ for the assessment of HMG-CoA reductase activities in the rats. The liver (0·2 g) samples were minced and homogenised in ice-cold 50 mm-Tris-HCl buffer (pH 7·5) and centrifuged at 1000 ***g*** at room temperature (23 ± 2°C) for 30 min. The pellets were discarded and the supernatant fractions were subjected to the detection of HMG-CoA reductase activity, if any. HMG-CoA reductase activities in liver homogenates were estimated spectrophotometrically from the ratio of HMG-CoA:mevalonate (H:M), according to the method reported by Rao & Ramakrishnan^(36)^.

### Histopathological studies and determination of liver and kidney markers

To confirm whether there are any morphological changes in livers and kidneys of the experimental rats, the said organs were examined under light microscopy (Olympus). The levels of different liver (SGPT (serum glutamic pyruvic transaminase) and SGOT (serum glutamic-oxaloacetic transaminase)) and kidney markers (BUN (blood urea N), serum urea and creatinine) were determined to ascertain toxic effects of the extracts on the said organs, if any^(32)^.

### Estimation of human equivalent dose

There are four different methods of extrapolation of dose from animals to humans, namely: dose by factor, similar drug, pharmacokinetically guided and comparative approaches^(37)^. In the present study, the ‘dose by factor’ approach has been used to estimate human equivalent dose (HED) values of SC_best_ and YM_best_ from their respective optimised animal doses.

### Molecular docking studies with extract components and 3-hydroxy-3-methyl-glutaryl-CoA reductase

The binding affinities of major components of SC_best_ and YM_best_ with HMG-CoA reductase were computed by molecular docking to identify the chemical compounds in the extracts which induced hypocholesterolaemia. The major components of the aforesaid extracts include limonene and linalool, as well as α-terpinyl acetate, α-terpineol and 1,8-cineole in SC_best_; and melatonin, tocopherol and ascorbic acid in YM_best_. Therefore, the aforesaid components were compared structurally with the substrate (HMG-CoA) and inhibitors (statins) of HMG-CoA receptor protein. Since melatonin reportedly does not possess inhibitory activity on HMG-CoA reductase as evidenced in Sprague–Dawley rat models^(38)^, docking of this biomolecule with HMG-CoA reductase was not performed.

Three-dimensional structures of extract components and HMG-CoA reductase were retrieved from the PubChem database^(39)^ and the solvent-accessible surface area of HMG-CoA reductase was generated using Chimera^(40)^. The top ten energetically preferred conformers of the above-mentioned biomolecules (except 1,8-cineole since it lacks rotatable bonds) were generated using FROG2 (FRee On line druG conformation generation) software^(41)^. Molecular docking of components (present in SC_best_ and YM_best_) was performed using one of the dimer structures of HMG-CoA receptor collected from the Protein Data Bank (PDB)^(42)^ (PDB ID: 1HWK). Initially, ingredient molecules were docked at the statin binding site of HMG-CoA receptor using the GOLD (Genetic Optimization for Ligand Docking) package^(43)^. GOLD software optimises the fitness score of many possible docking solutions using a genetic algorithm. The following parameters were used in the docking cycles: population size (10), selection pressure (1 100 000), number of operations (100 000), number of islands (5), niche size (2), crossover weight (95), mutate weight (95) and migrate weight (10). Docking solutions from various conformers were clustered based on their root mean square deviation (RMSD) representing structural similarity. Sub-clusters with a minimum of three solutions with RMSD <2 Å with each other were considered for further analysis. The top three scoring solutions (pose) from the largest cluster were selected for further comparison. For comparison purpose, rescoring of HMG-CoA (substrate) and statins (known inhibitors) of HMG-CoA receptor structures were carried out via the GOLD program. Five statin-bound structures (PDB ID: 1HW8, 1HW9, 1HWI, 1HWJ and 1HWL) and one HMG-CoA-bound HMG-CoA receptor structure (PDB ID: 1DQ9) were collected from the PDB.

We have also docked the active ingredient molecules at pockets other than the substrate/inhibitor binding sites. CASTp^(44)^ and metaPockets^(45)^ programs were used to predict pockets in the HMG-CoA receptor protein. Commonly predicted pockets were selected for further docking where each active ingredient was docked onto all these pockets and a similar protocol (as described before) was applied to identify the most likely solutions. Docking scores of most likely solutions were plotted and the ligand–protein interactions were identified by the LigPlot program^(46)^.

### Statistical analyses of biochemical parameters

The experimental results are expressed as means of the experimental data obtained from six rats in each group. Significant differences between mean values were determined by Student's *t* test and Duncan's multiple-range test using STATISTICA 8.0 software (Statsoft). A *P* value of ≤0·05 was used to verify the significance of the tests.

## Results

### Chemical profiling, quality and safety of YM_best_ and SC_best_

**Fig. 4.**
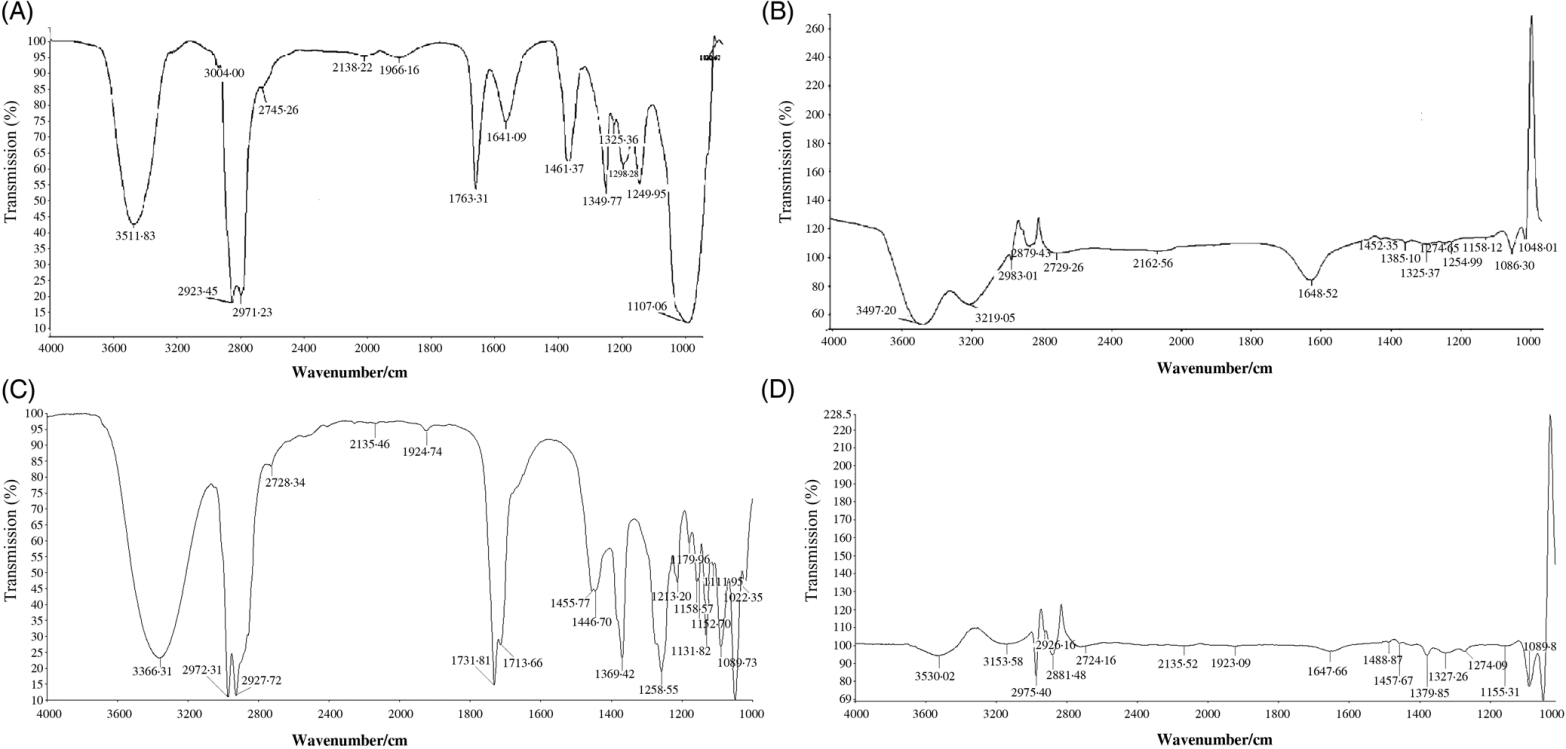
Fourier transformed IR spectroscopy spectra of (A) melatonin, (B) supercritical carbon dioxide (SC-CO_2_) extract of yellow mustard seeds, (C) 1,8-cineole and (D) SC-CO_2_ extract of small cardamom seeds.

**Fig. 5.**
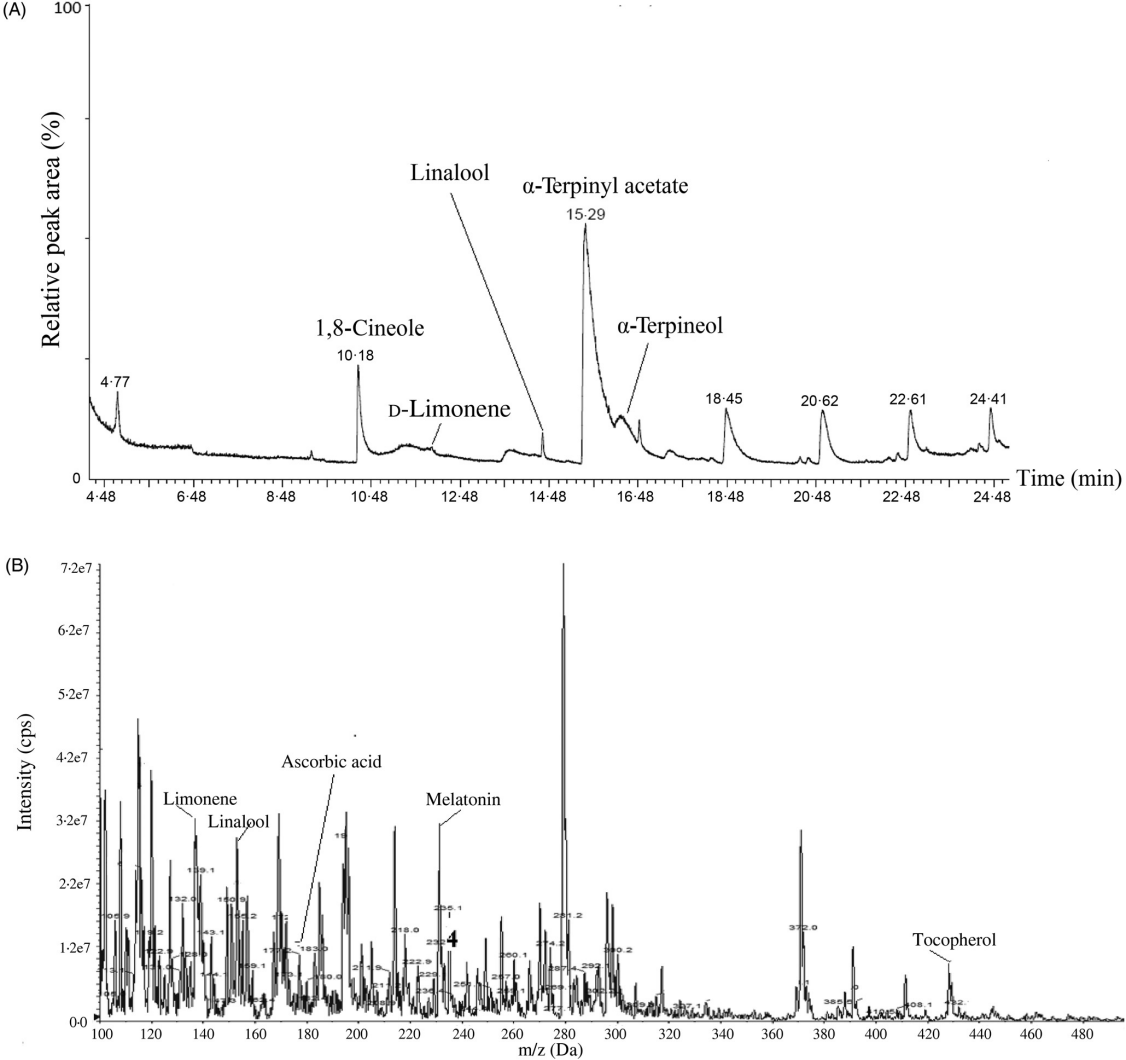
(A) GC-MS chromatogram of supercritical carbon dioxide (SC-CO_2_) extract of small cardamom seeds and (B) liquid chromatography-MS chromatogram of SC-CO_2_ extract of yellow mustard seeds. cps, Counts per s.

The FT-IR spectra of YM_best_ and SC_best_ were analysed using the data published by Dyer^(47)^. The wavenumbers in the FT-IR spectrum of SC_best_ (Fig. 4) confirmed the presence of 1,8-cineole and also indicated the presence of α-terpinyl acetate, limonene, α-terpineol and linalool^(48–51)^. The identities of these antioxidants were further confirmed by GC-MS analysis (Fig. 5). The relative peak percentage areas of 1,8-cineole, α-terpinyl acetate, limonene, α-terpineol and linalool in SC_best_ were 22·09, 30·65, 4·35, 6·42 and 0·40, respectively.

In the case of YM_best_, the presence of C=C (1463·8/cm), methoxy group (2854·55/cm) and five membered ring C–C multiple bond stretching (1744·12/cm) in the FT-IR spectrum (Fig. 4) confirmed the presence of melatonin therein. Peaks in the range of 3008/cm (unsaturated double bonds, =CH, *cis* bonds) and 1654/cm (–C=C–, *cis* bonds) suggested the presence of fatty acids in the extracts. The FT-IR spectra of standard oleic acid, limonene and tocopherol also suggested their presence in the extracts^(52)^. LC-ESI-MS (Fig. 5) analysis also confirmed the presence of the above co-extracted compounds in YM_best_.

YM_best_ and SC_best_ exhibited 62·89 and 83·87 % DPPH radical-scavenging activities, respectively, as revealed from their EPR spectra. The SE values of YM_best_ and SC_best_ were greater than unity for either extract, namely 1·09 and 1·16, respectively. EDX analyses revealed that no heavy metal was present in either extract.

### Acute toxicities of Triton X-100 and supercritical carbon dioxide extracts

The wellness parameters of rats are presented in Supplementary Table S1. Mortality was found in two rats treated with Triton X-100 (2000 mg/kg BW) within 24 h; however, the remaining three rats were without any signs of toxicity up to 48 h of dose administration.

The rats administered with YM_best_ and SC_best_ extracts (5000 mg/kg BW) did not show any sign of toxicity, such as salivation, lethargy, convulsions, tremors and diarrhoea within 48 h of dose administration (Supplementary Tables S2 and S3). Therefore, the LD_50_ value of Triton X-100 was below 2000 mg/kg BW, whereas the same for SC-CO_2_ extracts were greater than 5000 mg/kg BW.

### Standardised dose of Triton X-100 for inducing hypercholesterolaemia

Administration of Triton X-100 at multiple doses for 3 consecutive days did not result in a stable serum TC level (Supplementary Fig. S1). In addition, it led to severe structural deterioration in liver and kidney cells, such as damaged glomeruli and dilated renal tubules which in turn impede cholesterol biosynthesis in liver. Similar deterioration in the structure of kidneys was also observed in a toxicity study on acetaminophen^(32)^.

However, a single dose of Triton X-100 (175 mg/kg BW) could maintain a stable hypercholesterolaemic condition without any significant histological alteration (in the structure of kidney and liver cells) after 72 h of administration. Therefore, based on serum TC level and histological studies, a single dose of 175 mg/kg BW of Triton X-100 was considered to be the optimised dose for inducing hypercholesterolaemia in rats.

### *In vivo* efficacies of SC_best_ and YM_best_ on hypercholesterolaemic rats

#### Effects on serum total cholesterol levels

**Fig. 6.**
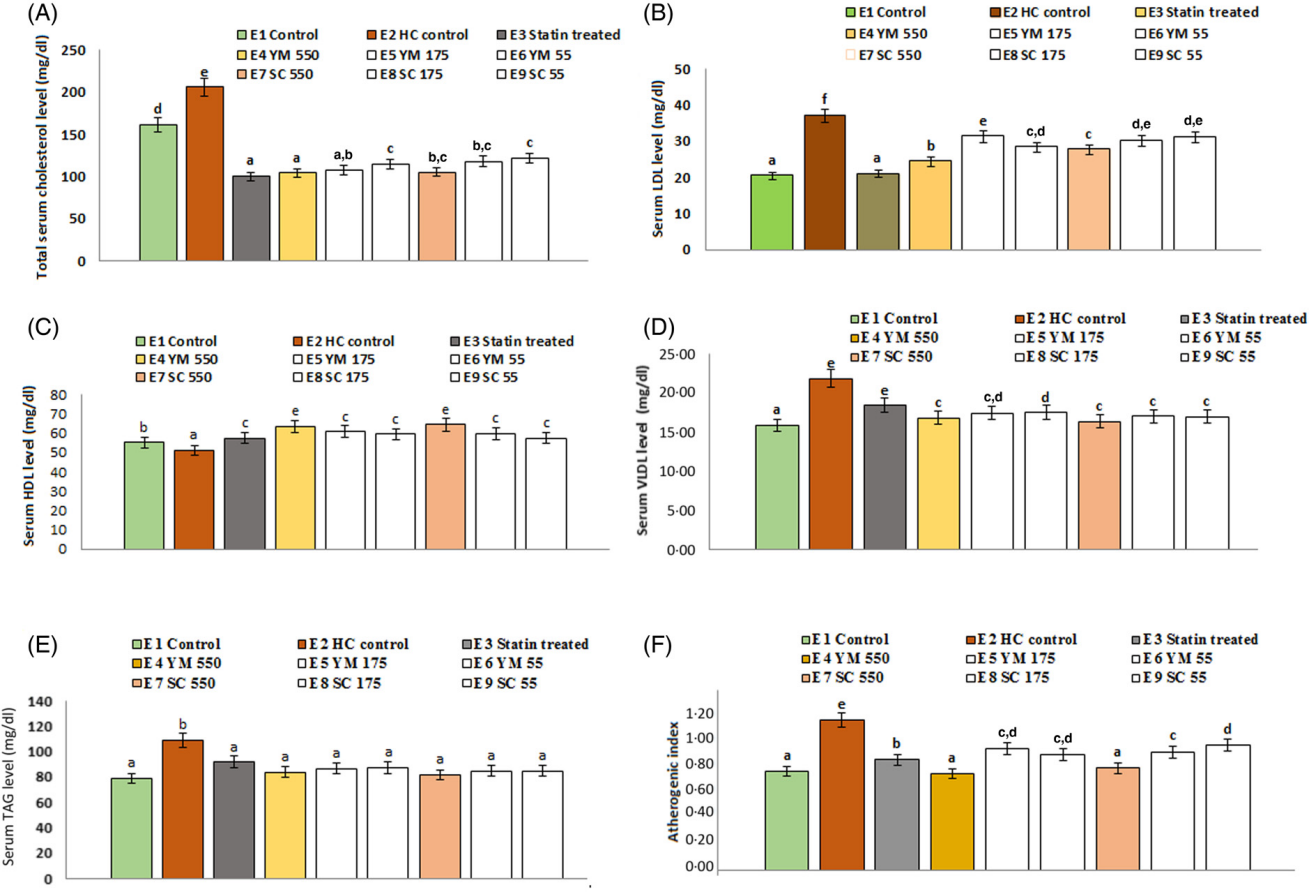
Effects of supercritical carbon dioxide extracts of yellow mustard (YM) and small cardamom (SC) seeds on lipid profiles of Wistar albino rats. Values are means, with standard deviations represented by vertical bars. ^a,b,c,d^ Mean values with unlike letters were significantly different (*P* < 0·05; Duncan's multiple-range test). To convert cholesterol in mg/dl to mmol/l, multiply by 0·0259. To convert TAG in mg/dl to mmol/l, multiply by 0·0113.

The effects of SC_best_ and YM_best_ on serum TC levels of rats are shown in Fig. 6. Among three different doses (55, 175, 550 mg/kg BW) of extracts, the maximum reduction in serum TC level (49·44 (sd 3·30) % and 48·95 (sd 2·50) % by YM_best_ and SC_best_, respectively) was found at 550 mg/kg BW on day 15. Further feeding of either extract up to day 21 did not cause any significant (*P* = 0·000 in either case) change in serum TC level. Percentage reduction in serum TC level was maximum in atorvastatin (51·26 (sd 4·30) %) treated rats, which corroborated well with the findings of Pasha^(53)^. No adverse effects in the animals were found during the experimental period.

#### Effect on serum TAG levels

Administration of YM_best_ and SC_best_ at 550 mg/kg BW and atorvastatin (10 mg/kg BW) individually resulted in significant lowering (*P* = 0·000 in all the cases) of serum TAG levels compared with those in healthy control rats (Fig. 6) on day 21. The decrease in TAG levels of YM_best_ (22·89 (sd 2·58( %) and SC_best_ (25·15 (sd 1·11) %) treated rats were found to be higher than atorvastatin-treated rats (15·74 (sd 1·04) %).

#### Effects on serum HDL-cholesterol, LDL-cholesterol and VLDL-cholesterol levels and atherogenic index values

The rats treated with YM_best_ and SC_best_ (groups E_4_ and E_7_) showed significant increases (*P* = 0·008 and 0·033, respectively) in serum mean HDL-C levels with concomitant decreases in serum mean LDL-C (*P* = 0·000 in either case) levels compared with those in the control group of rats (group E_2_) on day 21 (Fig. 6). Additionally, the serum mean VLDL-cholesterol levels (0·48 mmol/l; 18·37 mg/dl) also decreased on day 21, in statin- and extract-treated rats (0·44  (sd  0·04)–0·42  (sd  0·06) mmol/l; 16·81  (sd  1·45)–16·32  (sd  2·34 mg/dl) compared with those in healthy control rats (0·57  (sd  0·06) mmol/l; 21·84 (sd 0·36) mg/dl). AI values (0·99 (sd 0·08)) of healthy control rats were significantly higher (*P* = 0·000) than those of the extract (0·68 (sd 0·03) to 0·80 (sd 0·05)) and atorvastatin-treated (0·69 (sd 0·05)) rats (Fig. 6).

#### Effects on serum total cholesterol levels after discontinuation of administration of supercritical carbon dioxide extracts

**Fig. 7.**
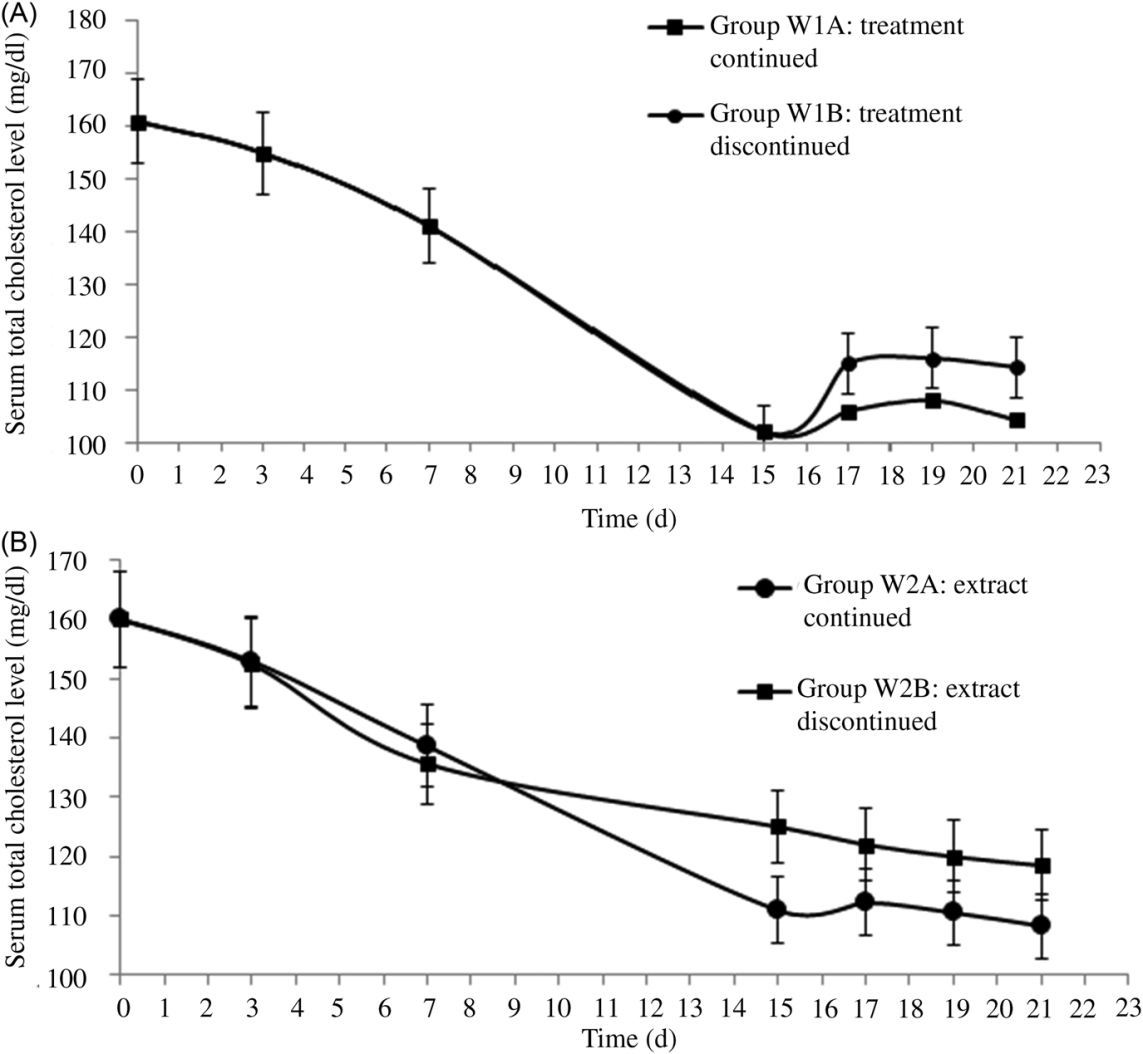
Serum cholesterol levels after discontinuation of treatment with (A) yellow mustard seed extract and (B) small cardamom seed extract. Values are means, with standard deviations represented by vertical bars. To convert cholesterol in mg/dl to mmol/l, multiply by 0·0259.

Rats of group W_1A_ and W_1B_ (YM_best_ treated) showed mean serum TC levels of 0·48  (sd  0·13) mmol/l (104·28 (sd 5·00) mg/dl) and 2·97  (sd  0·18) mmol/l (114·56 (sd 6·96 mg/dl), respectively. The rats of W_2A_ and W_2B_ (SC_best_ treated) showed serum TC levels of 2·80  (sd  0·13) mmol/l (108·29 (sd 4·89) mg/dl) and 3·07  (sd  0·17) mmol/l (118·34 (sd 6·65) mg/dl), respectively (Fig. 7). In both cases, the differences between the serum TC levels of treatment continued and discontinued groups were insignificant (*P* = 0·041 for YM_best_-administered rats; *P* = 0·022 for SC_best_-administered rats) on day 21. The present study conclusively suggests that the extracts could be served for 15 d at a dose of 550 mg/kg BW to achieve a normal serum TC level in hypercholesterolaemic rats.

#### Effects on hepatic 3-hydroxy-3-methyl-glutaryl-CoA reductase activity

On day 21, hepatic HMG-CoA:mevalonate ratios also increased in YM_best_ (2·70 (sd 0·12)), SC_best,_ (2·76 (sd 0·19)) and atorvastatin (2·86 (sd 0·32)) treated rats than those of healthy control rats (2·33 (sd 0·09)) (Table 1), indicating possible enzyme-inhibitory effect of the extracts.

**Table 1.** Effect of supercritical carbon dioxide extracts of yellow mustard seeds and small cardamom seeds on 3-hydroxy-3-methyl-glutaryl-CoA:mevalonate (H:M) ratio, kidney and liver markers in hypercholesterolaemic rats (Mean values and standard deviations; six rats per group)

		H:M ratio		BUN (mg/dl)*		Creatinine (mg/dl)*		Urea (mg/dl)*		SGOT (IU/l)		SGPT (IU/l)	
Groups	Description of animal groups	Mean	sd	Mean	sd	Mean	sd	Mean	sd	Mean	sd	Mean	sd
E_1_	Regular oral administration of 10 % DMSO solution for 21 d	2·95^a^	0·12	18·01^a^	0·91	0·60^a^	0·02	33·29^a^	1·33	40·78^a^	2·11	40·00^a^	2·11
E_2_	Injected intraperitoneally with single dose of Triton X-100 (175 mg/kg BW) on day 1 and regular oral administration of 10 % DMSO solution for 21 d	2·33^b^	0·09	23·00^b^	0·88	0·97^b^	0·03	67·19^b^	2·31	89·47^b^	1·99	51·25^b^	1·99
E_3_	Injected intraperitoneally with single dose of Triton X-100 (175 mg/kg BW) on day 1 and oral administration of atorvastatin 10 mg/kg BW for 21 d	2·86^a^	0·32	18·21^a^	0·98	0·75^c^	0·03	35·26^a^	1·88	42·45^a^	5·01	39·20^a^	5·01
E_4_	Injected intraperitoneally with single dose of Triton X-100 (175 mg/kg BW) on day 1 and oral administration of YM_best _at the dose of 550 mg/kg BW for 21 d	2·42^c^	0·11	19·45^a^	0·89	0·78^c^	0·01	41·25^c^	1·71	45·02^a^	4·89	39·50^a^	4·89
E_5_	Injected intraperitoneally with single dose of Triton X-100 (175 mg/kg BW) on day 1 and oral administration of YM_best _at the dose of 175 mg/kg BW for 21 d	2·42^d^	0·15	22·08^a^	0·92	0·88^c^	0·04	50·00^d^	2·05	46·69^a^	6·88	42·29^a^	6·88
E_6_	Injected intraperitoneally with single dose of Triton X-100 (175 mg/kg BW) on day 1 and oral administration of YM_best _at the dose of 55 mg/kg BW for 21 d	2·70^d^	0·12	20·02^a^	0·99	0·92^b^	0·05	61·66^d^	1·98	48·12^a^	2·73	41·36^a^	2·73
E_7_	Injected intraperitoneally with single dose of Triton X-100 (175 mg/kg BW) on day 1 and oral administration of SC_best _at the dose of 550 mg/kg BW for 21 d	2·55^c^	0·16	19·00^a^	0·83	0·85^c^	0·04	40·23^c^	1·59	44·14^a^	4·56	40·05^a^	4·56
E_8_	Injected intraperitoneally with single dose of Triton X-100 (175 mg/kg BW) on day 1 and oral administration of SC_best _at the dose of 175 mg/kg BW for 21 d	2·66^d^	0·08	21·02^a^	0·94	0·81^c^	0·03	51·44^d^	2·13	46·08^a^	6·32	42·85^a^	6·32
E_9_	Injected intraperitoneally with single dose of Triton X-100 (175 mg/kg BW) on day 1 and oral administration of SC_best _at the dose of 55 mg/kg BW for 21 d	2·76^c^	0·19	20·00^a^	0·89	0·83^c^	0·05	63·04^d^	1·34	48·99^a^	2·18	42·77^a^	2·18

BUN, blood urea N; SGOT, serum glutamic-oxaloacetic transaminase; SGPT, serum glutamic pyruvic transaminase; DMSO, dimethylsulfoxide; BW, body weight; YM_best_, yellow mustard seed extract obtained at the optimum conditions of supercritical CO_2_; SC_best_, small cardamom seed extract obtained at the optimum conditions of supercritical CO_2_.

^a,b,c,d^ Mean values within a column with unlike superscript letters were significantly different (*P* < 0·05; Duncan's multiple-range test).

* To convert BUN from mg/dl to mmol/l, multiply by 0·357. To convert creatinine from mg/dl to μmol/l, multiply by 88·4. To convert urea from mg/dl to mmol/l, multiply by 0·1665.

#### Histological changes and effects on liver and kidney markers

**Fig. 8.**
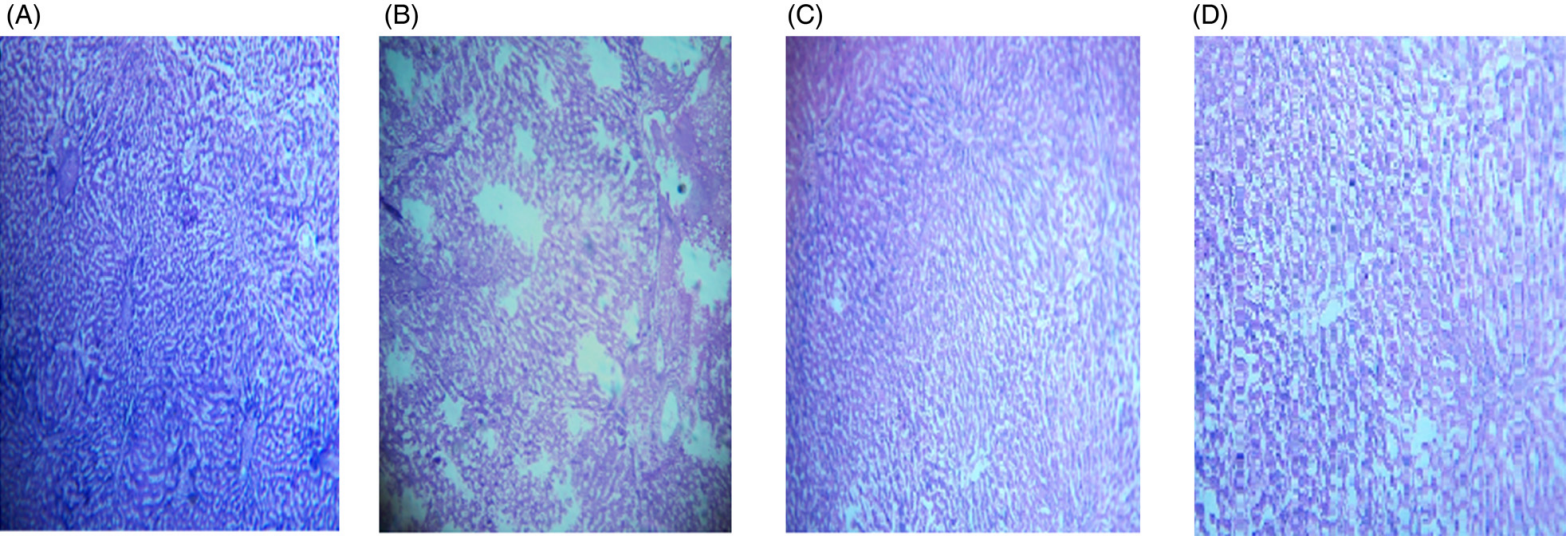
Histology of liver of (A) control group of rats showing well-organised lobular structure, (B) group of rats treated with optimised dose of Triton X-100 (175 mg/kg body weight (BW)) showing disorganised structure of liver, (C) rats treated with yellow mustard seed extract (550 mg/kg BW) and (D) small cardamom seed extract (550 mg/kg BW) showing organised structure of hepatic lobules.

**Fig. 9.**
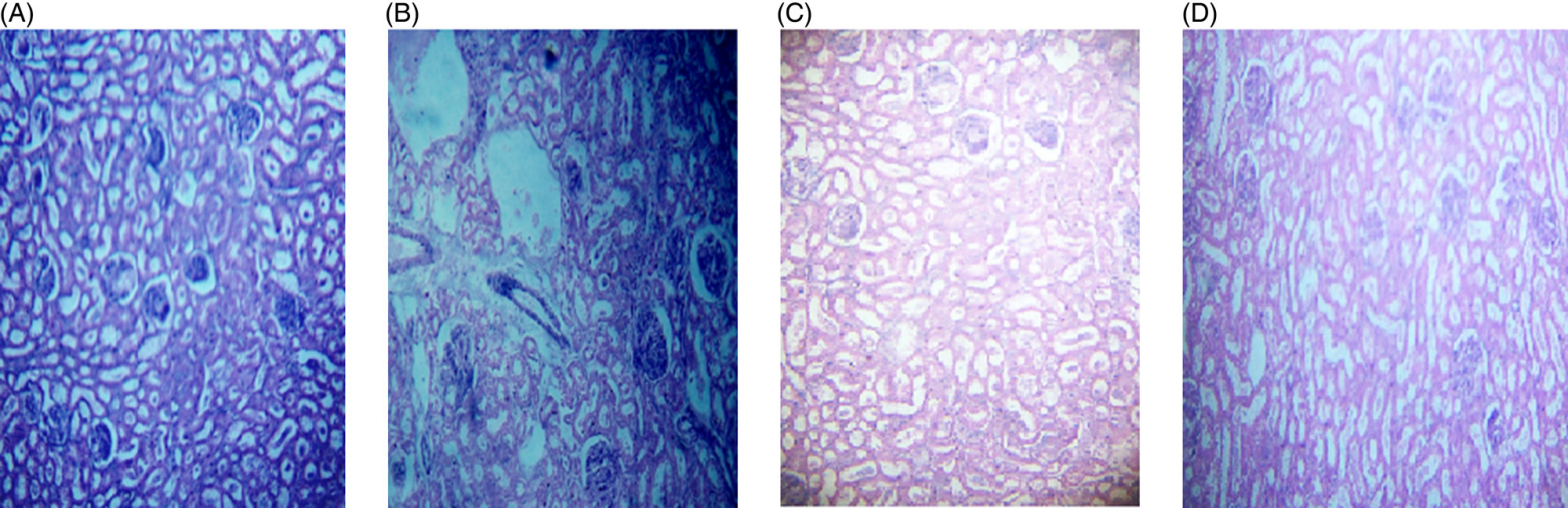
Histology of kidney of (A) control group of rats showing normal renal tubules with intact well-organised cellular boundary, (B) rats treated with multiple doses of Triton X-100 (175 mg/kg body weight (BW)) showing severe disorganisation in the renal tubules, (C) rats treated with yellow mustard seed extract (550 mg/kg BW) and (D) small cardamom seed extract (550 mg/kg BW) showing normal organisation in the renal tubules.

The microscopic images of livers and kidneys are presented in Figs 8((A)–(D)) and 9((A)–(D)), respectively. There were significant changes in kidneys and livers of the Triton X-100-treated rats (175 mg/kg BW) on day 21. The liver of the extract-administered rats exhibited normal structure of central vein and hepatic cords (Fig. 8). Histological structures of kidneys of the hypocholesterolaemic control group (group E_2_) of rats showed severe disorganisation of the glomerulus and dilation of renal tubules; however, kidneys of extract-administered rats showed no such morphological aberrations (Fig. 9).

The levels of serum BUN (blood urea N), urea and creatinine (liver markers), serum SGPT (serum glutamic pyruvic transaminase) and SGOT (serum glutamic-oxaloacetic transaminase) (kidney markers) levels of the rats are presented in Table 1. No alternations in serum urea and creatinine levels were observed in the rats treated with atorvastatin and YM_best_ and SC_best_ during the experimental period of 21 d; however, the levels changed significantly (*P* = 0·000) in the healthy control (group E_2_) rats. Considering the animal dose to be 550 mg/kg BW at which no adverse effects in the animals were found, HED was calculated to be 89 mg/kg BW for SC_best_ and YM_best_.

### Docking scores of extract components with 3-hydroxy-3-methyl-glutaryl-CoA reductase

**Fig. 10.**
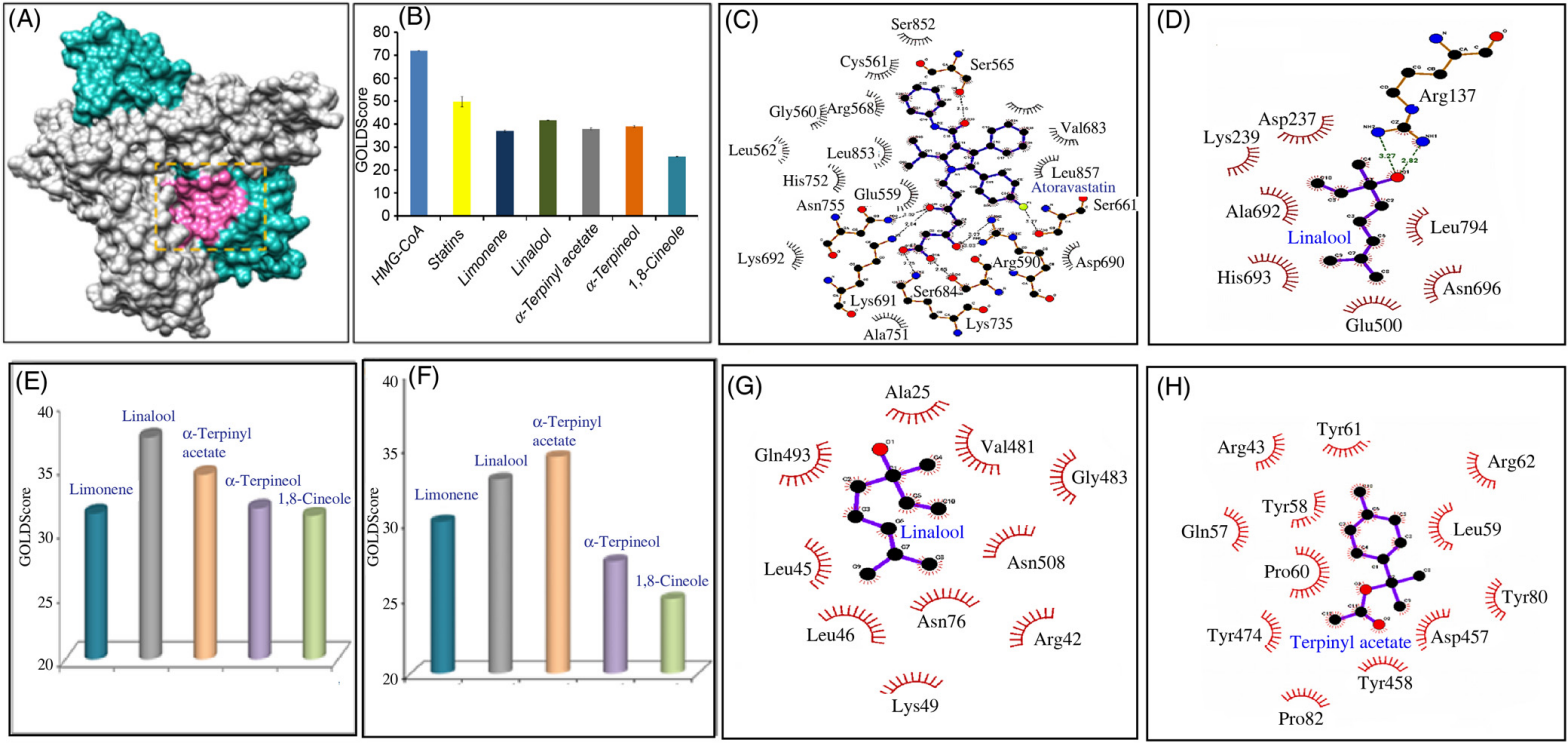
(A) Three-dimensional surface view of one of the dimer structures of 3-hydroxy-3-methyl-glutaryl-CoA (HMG-CoA) receptor (Protein Data Bank ID: 1HWK) where two chains are marked in grey and cyan and the substrate/inhibitor binding pocket is shown in pink. (B) Average molecular docking score represented as GOLDScore is plotted for each active ingredient of the cardamom extract when docked at the substrate/inhibitor binding site. GOLD derived rescores of the substrate (HMG-CoA) and inhibitors (statins) bound to HMG-CoA receptor protein are also plotted for comparison purposes. Values are means, with standard deviations represented by vertical bars. (C, D) LigPlot representations of the interacting residues and the probable interaction types for one of the known inhibitor (atorvastatin) and the highest-scoring active ingredient (linalool), respectively. (E, F) Average molecular docking scores represented as GOLDScore are plotted for each active ingredient of the cardamom extract when docked at the predicted binding sites, consensus pocket 2 and 3, respectively. (G, H) LigPlot representations of the interacting residues and the probable interaction types derived from linalool and α-terpinyl acetate docked at the predicted binding sites, consensus pocket 2 and 3, respectively.

Fig. 7 provides the docking scores of α-terpinyl acetate, α-terpineol, limonene, linalool and 1,8-cineole with respect to the bound substrate (HMG-CoA) and inhibitors (statins) of HMG-CoA receptor protein. Ingredient molecules were docked at the statin binding site (Fig. 10(A)) of HMG-CoA receptor using the GOLD package^(43)^. Average docking scores of the top three scoring solutions (pose) from the largest cluster of each active ingredient molecules were calculated and compared against GOLD-generated rescores derived from the substrate (HMG-CoA) and inhibitors (statins) bound to HMG-CoA receptor protein structures (Fig. 10(B)). Fig. 10(C) and 10(D) provide the LigPlot representation of the interacting residues and the probable interaction types for one of the known inhibitors (atorvastatin) and the highest scoring active ingredient (linalool), respectively.

The docking exercise at the statin-binding site of HMG-CoA reductase suggested that linalool and α-terpineol achieve docking scores comparable with therapeutically used HMG-CoA reductase inhibitors. However, the size and surface area of the active molecules are considerably smaller than the substrate HMG-CoA and the statins (Supplementary Fig. S2). Hence, they do not occupy the full substrate/inhibitor binding pocket, and instead bind to sub-pockets within the substrate/statin binding pocket.

The possibility of binding to other probable cavity/pockets was also explored via the prediction of pockets and subsequent docking to those pockets. Two such pockets were identified that were commonly predicted by two programs (Fig. S3). Each active ingredient was docked onto all these pockets and docking scores of the most likely solutions were plotted (Fig. 10(E) and 10(F)) and the ligand–protein interactions were identified by the LigPlot program (Fig. 10(G) and 10(H)). The docking exercise at those pockets also suggested that linalool and α-terpinyl acetate possess higher docking probabilities.

## Discussion

### Antioxidant synergy by reductionism

The green extraction technology of SC-CO_2_ has delivered ‘melatonin-rich YM_best_' and ‘1,8-cineole-rich SC_best_’ extracts from YM and SC seeds, respectively. Although reductionism through SC-CO_2_ extraction was conducted using statistical design models and analytical tools, chemical profiling and determination of SE values revealed a synergistic consortium of antioxidants in either extract, and not melatonin and 1,8-cineole as isolated single antioxidants. The present study established methodological reductionism as a successful approach to obtain consortia of bioactives from plant parts which exhibited antioxidant synergies. These synergistic consortia of antioxidants in the spices could reduce *in vivo* lipid peroxidation rate, restore antioxidant activity and delay the development of hypercholesterolaemia^(54)^ in animals, rendering the extracts suitable for *in vivo* investigation in redressing this oxidative stress disorder^(55)^.

### *In vivo* efficacies of the supercritical carbon dioxide extracts

Co-administration of YM_best_ and SC_best_ at a dose of 550 mg/kg BW for 15 d along with a single dose (175 mg/kg BW) of Triton X-100 resulted in the maximum reduction of serum TC levels. Hussain^(56)^ had reported a 13·75 % reduction in the serum TC level of hypercholesterolaemic Sprague–Dawley rats, treated with standard melatonin (10 mg/kg) for 30 d. However, a higher hypocholesterolaemic efficacy of YM_best_ extract has been established in the present study compared with those treated with whole mustard seeds (14·90 % after 90 d)^(16)^. Similarly, the rats treated with SC_best_ also exhibited a higher reduction in serum TC level than those reported by Nagashree *et al*.^(17)^ for SC seed oil (31 % reduction at a dose of 3 g/kg BW after 8 weeks). Therefore, it could be reasonably concluded that the strong hypocholesterolaemic activities of SC_best_ and YM_best_ were not owing to their respective bioactive principles alone but rather due to the consortia of bioactives therein. Moreover, administration of extracts also improved the serum TAG as well as lipoprotein levels (increase in HDL-C and decrease in LDL-C).

The HED of SC_best_ was found to be 89 mg/kg BW, which is higher than the reported dose (50 mg/kg BW) of whole cardamom (variety not reported) in clinical studies^(3,4)^. Since SC_best_ and YM_best_ are non-toxic and safe for consumption, either extract can be subjected to clinical trials at a HED of 89 mg/kg BW.

### Inhibitory effects of extract components on 3-hydroxy-3-methyl-glutaryl-CoA reductase in terms of docking scores

The inhibitory effects of the extracts on hepatic HMG-CoA reductase activities possibly contributed to the maintenance of normal serum TC and lipoprotein levels in hypercholesterolaemic rats. Molecular docking results revealed that linalool achieved the highest docking scores in the statin-bound site, suggesting its strong inhibitory action on HMG-CoA reductase. Both extracts therefore exhibited their inhibitory effects on hepatic HMG-CoA reductase activities owing to the presence of linalool therein. Additionally, α-terpineol and α-terpinyl acetate of SC_best_ are more potent in inhibiting HMG-CoA reductase activity than 1,8-cineole and limonene in terms of their respective docking scores.

Since melatonin has no inhibitory effect on HMG-CoA reductase activity, the docking study suggested that the said activity of the YM_best_ extract is conferred possibly by limonene and linalool therein. Additionally, *in vivo* studies reported by several authors suggested that other major co-extractants of YM_best_, namely tocopherol^(57)^, ascorbic acid^(58)^ and MUFA (chiefly arachidic acid, palmitic acid, caproic acid, caprylic acid)^(59)^, possess inhibitory efficacies on HMG-CoA reductase activity. Thus, it could be reasonably concluded that all the major co-extractants in YM_best_ extract including melatonin contributed in maintaining the serum TC levels by inhibiting HMG-CoA reductase activity.

Although melatonin has no inhibitory effect on HMG-CoA reductase activity, augmenting effects of melatonin on lecithin-cholesterol acyltransferase (LCAT)-mediated cholesterol esterification (leading to increased synthesis of HDL-C) and decreased TC level, have been reported by Esquifino *et al*.^(60)^. From the above studies, it can be unambiguously concluded that linalool, α-terpineol and α- terpinyl acetate in SC_best_ and linalool (besides tocopherol, ascorbic and MUFA) in YM_best_ inhibited HMG-CoA reductase activity contributing to lowering of serum TC level in rats.

### Authentic natural leads and not invalid metabolic panaceas

It is always important to validate the authenticity of bioactive principles in natural resources prior to designating them as natural leads to avoid invalid metabolic panaceas of the compounds. Although molecular docking of the respective bioactive compounds of both extracts indicated their individual inhibitory effects on HMG-CoA reductase activity, they may not possess the same property when present in combination in the extracts, if they do not act in synergism with other compounds therein. Moreover, they may impede bioactivities of the other co-extractants. *In silico* molecular docking and *in vivo* hypocholesterolaemic studies revealed that oral administrations of YM_best_ and SC_best_ inhibited hepatic HMG-CoA reductase activities and therefore possibly down-regulated *de novo* cholesterol synthesis in the rats. These biotherapeutic molecules are therefore not invalid metabolic panaceas but authentic natural leads for the design of therapeutic foods and natural product-based drugs.

### Conclusion

In the present study, it can be reasonably concluded that the consortia of bioactives in YM_best_ and SC_best_ conferred the SC-CO_2_ extracts as novel spice-based biotherapeutics in preventing hypercholesterolaemia and therefore cannot be considered as invalid metabolic panaceas. Oral administration of extracts in rats would exhibit dual benefits, namely the inhibition of HMG-CoA reductase activity resulting in the prevention of *de novo* cholesterol synthesis, and scavenging of reactive oxygen species leading to reduction of hypercholesterolaemia-induced oxidative stress. These ‘green’ extracts therefore qualify for human clinical trials and could be safely consumed *per se* as hypocholesterolaemic supplements. These SC-CO_2_ extracts could also be utilised in designing new therapeutic foods for redressing hypercholesterolaemia.

## Supplementary material

For supplementary material accompanying this paper visit https://doi.org/10.1017/jns.2019.28. click here to view supplementary material
