# Supplementary material for: Consortia of bioactives in supercritical carbon dioxide extracts of mustard and small cardamom seeds lower serum cholesterol levels in rats: new leads for hypocholesterolaemic supplements from spices
Source: J Nutr Sci. 2019 Sep 24;8:e32. doi: 10.1017/jns.2019.28 (PMC6764189; doi:10.1017/jns.2019.28)
Supplement: Supplementary file 1 [file S2048679019000284sup.zip › S2048679019000284sup001.docx]

| **Supplementary Table S1.** Observation of wellness parameters of rats for the limit test of Triton-X 100 at the dosage rate of 2000 mg/kg body weight | | | | | | | | | | | | | | |
| --- | --- | --- | --- | --- | --- | --- | --- | --- | --- | --- | --- | --- | --- | --- |
| Observation | Observation for the test at 2000 mg/kg b.w. of rats (h) | | | | | | | | | | | | | |
|  | 0.5 h | | 4h | | | 12h | | | 24h | | | 48h | | |
|  | C | E | | C | E | | C | E | | C | E | | C | E |
| Skin and fur | Normal | Normal | | Normal | Normal | | Normal | Normal | | Normal | 2X3L | | Normal | 3X2L |
| Eyes | Normal | Normal | | Normal | Normal | | Normal | Normal | | Normal | 2X3L | | Normal | 3X2L |
| Mucous membrane | Normal | Normal | | Normal | Normal | | Normal | Normal | | Normal | 2X3L | | Normal | 3X2L |
| Salivation | No | No | | No | No | | No | No | | No | 2X3L | | No | 3X2L |
| Lethargy | No | No | | No | No | | No | Yes | | No | 2X3L | | No | 3X2L |
| Sleep | Normal | Normal | | Normal | Normal | | Normal | Normal | | Normal | 2X3L | | Normal | 3X2L |
| Convulsions | No | No | | No | No | | No | No | | No | 2X3L | | No | 3X2L |
| Tremors | No | No | | No | Yes | | No | No | | No | 2X3L | | No | 3X2L |
| Diarrhoea | No | No | | No | No | | No | No | | No | 2X3L | | No | 3X2L |
| Morbidity | No | No | | No | No | | No | No | | No | 2X3L | | No | 3X2L |
| Mortality | No | No | | No | No | | No | No | | No | 2X3L | | No | 3X2L |

C: Control, E: Experimental, 2X3L: 2 Expired 3 live, 3X2L: 3 Expired 2 Live

| **Supplementary Table S2.** Observation of wellness parameters of rats for the limit test of YM seed extract at the dosage rate of 2000 mg/kg body weight | | | | | | | | | | |
| --- | --- | --- | --- | --- | --- | --- | --- | --- | --- | --- |
| Observation | Observation for the test at 2000 mg/kg b.w. of rats (h) | | | | | | | | | |
|  | 0.5 h | | 4h | | 12h | | 24h | | 48h | |
|  | C | E | C | E | C | E | C | E | C | E |
| Skin and fur | Normal | Normal | Normal | Normal | Normal | Normal | Normal | Normal | Normal | Normal |
| Eyes | Normal | Normal | Normal | Normal | Normal | Normal | Normal | Normal | Normal | Normal |
| Mucous membrane | Normal | Normal | Normal | Normal | Normal | Normal | Normal | Normal | Normal | Normal |
| Salivation | No | No | No | No | No | No | No | No | No | No |
| Lethargy | No | Yes | No | No | No | No | No | No | No | No |
| Sleep | Normal | Normal | Normal | Normal | Normal | Normal | Normal | Normal | Normal | Normal |
| Convulsions | No | No | No | No | No | No | No | No | No | No |
| Tremors | No | No | No | No | No | No | No | No | No | No |
| Diarrhoea | No | No | No | No | No | No | No | No | No | No |
| Morbidity | No | No | No | No | No | No | No | No | No | No |
| Mortality | No | No | No | No | No | No | No | No | No | No |

C: Control, E: Experimental

| **Supplementary Table S3.** Observation of wellness parameters of rats for the limit test of SC seed extract at the dosage rate of 2000 mg/kg body weight | | | | | | | | | | | |
| --- | --- | --- | --- | --- | --- | --- | --- | --- | --- | --- | --- |
| Observation | Observation for the test at 2000 mg/kg b.w. of rats (h) | | | | | | | | | |  |
|  | 0.5 h | | 4h | | 12h | | 24h | | 48h | |  |
|  | C | E | C | E | C | E | C | E | C | E |  |
| Skin and fur | Normal | Normal | Normal | Normal | Normal | Normal | Normal | Normal | Normal | Normal |  |
| Eyes | Normal | Normal | Normal | Normal | Normal | Normal | Normal | Normal | Normal | Normal |  |
| Mucous membrane | Normal | Normal | Normal | Normal | Normal | Normal | Normal | Normal | Normal | Normal |  |
| Salivation | No | No | No | No | No | No | No | No | No | No |  |
| Lethargy | No | No | No | No | No | No | No | No | No | No |  |
| Sleep | Normal | Normal | Normal | Normal | Normal | Normal | Normal | Normal | Normal | Normal |  |
| Convulsions | No | No | No | No | No | No | No | No | No | No |  |
| Tremors | No | No | No | No | No | No | No | No | No | No |  |
| Diarrhoea | No | No | No | No | No | No | No | No | No | No |  |
| Morbidity | No | No | No | No | No | No | No | No | No | No |  |
| Mortality | No | No | No | No | No | No | No | No | No | No |  |

C: Control, E: Experimental
