# Supplementary material for: Consortia of bioactives in supercritical carbon dioxide extracts of mustard and small cardamom seeds lower serum cholesterol levels in rats: new leads for hypocholesterolaemic supplements from spices
Source: J Nutr Sci. 2019 Sep 24;8:e32. doi: 10.1017/jns.2019.28 (PMC6764189; doi:10.1017/jns.2019.28)
Supplement: Supplementary file 1 [file S2048679019000284sup.zip › S2048679019000284sup002.docx]

**Supplementary material: Supplementary figure legends**


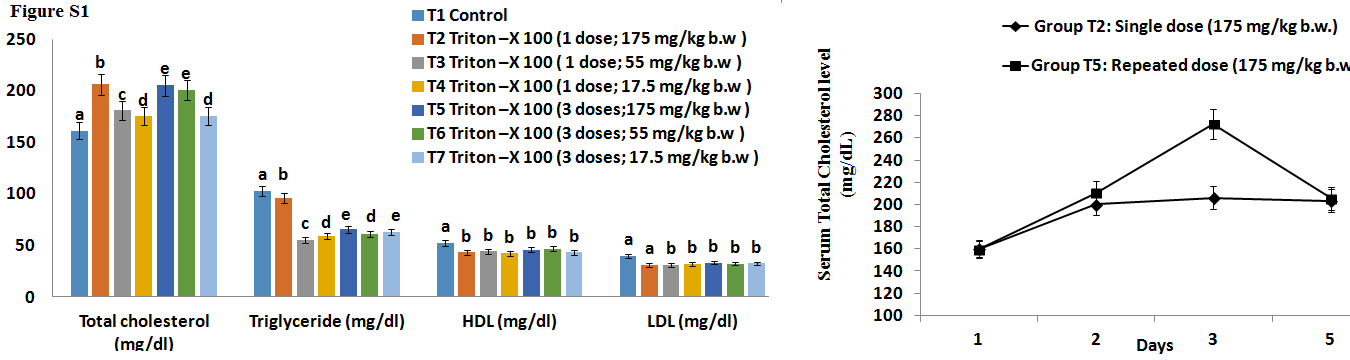


**Supplementary Fig. S1.** Effects of administration of Triton X-100 at single as well as multiple doses on serum TC level of the rats.

**
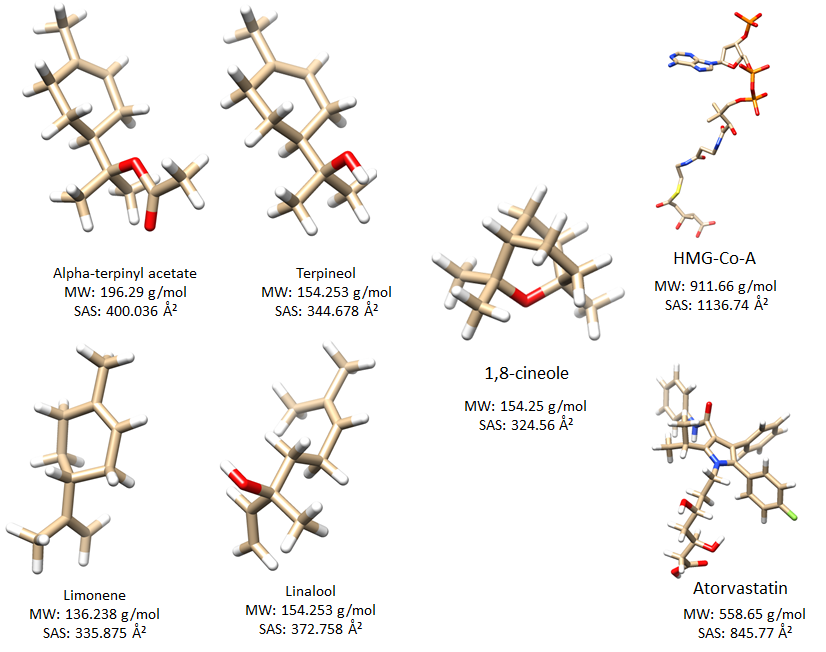
**

**Supplementary Fig. S2.** Molecular size and surface area of the active molecules cardamom extract. (MW: molecular weight; SAS: solvent accessible surface area).


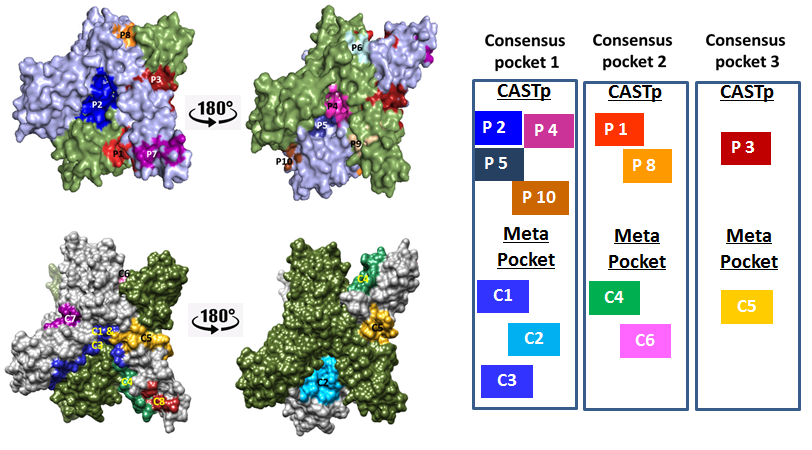


**Supplementary Fig. S3.** Predicted binding pockets of HMG-CoA receptor. CASTp and MetaPocket predicted pockets are mapped onto the 3D surface of the HMG-CoA receptor protein. CASTp and MetaPocket predicted pockets that are located at the similar region and overlaps significantly are considered consensus pockets.
